# Electrochemical, spectroscopic and theoretical monitoring of anthracyclines’ interactions with DNA and ascorbic acid by adopting two routes: Cancer cell line studies

**DOI:** 10.1371/journal.pone.0205764

**Published:** 2018-10-29

**Authors:** Fouzia Perveen, Nasima Arshad, Rumana Qureshi, Jahanzaib Nowsherwan, Aiesha Sultan, Bushra Nosheen, Hummera Rafique

**Affiliations:** 1 Research Centre for Modeling and Simulation, National University of Science and Technology, Islamabad, Pakistan; 2 Department of Chemistry, Allama Iqbal Open University, Islamabad, Pakistan; 3 Department of Chemistry, Quaid-i-Azam University, Islamabad, Pakistan; 4 Department of Chemistry, University of Gujrat, Gujrat, Pakistan; Aligarh Muslim University, INDIA

## Abstract

Pharmacodynamic interactions of three anthracycline antibiotics namely doxorubicin (DXH), epirubicin (EpiDXH) and daunorubicin (DNR) with DNA in the absence and presence of ascorbic acid (AA) as natural additive were monitored under physiological conditions (pH = 7.4, 4.7 and T = 309.5K). Route–1 (Anthracycline–AA–DNA) and Route–2 (Anthracycline–DNA–AA) were adopted to see the interactional behavior by cyclic voltammetry (CV) and UV-visible spectroscopy. In comparison to Route–2; voltammetric and spectral responses as well as binding constant (*K*_*b*_) and Gibb’s free energy change (Δ*G*) values revealed strongest and more favorable interaction of anthracycline–AA complex with DNA via Route–1. *K*_*b*_, *s* (binding site sizes) and Δ*G* evaluated from experimental (CV, UV-Vis) and theoretical (molecular docking) findings showed enhanced binding strength of tertiary complexes as compared to binary drug–DNA complexes. The results were found comparatively better at pH 7.4. Consistency was observed in binding parameters evaluated from experimental and theoretical techniques. Diffusion coefficients (*D*_*o*_) and heterogeneous electron transfer rate constant (*k*_*s*,*h*_) confirmed the formation of complexes via slow diffusion kinetics. Percent cell inhibition (%C_inh_) of anthracyclines for non-small cell cancer cell lines (NSCCLs) H-1299 and H-157 were evaluated higher in the presence of AA which further complimented experimental and theoretical results.

## 1. Introduction

The clinical importance of anthracycline antibiotics is obvious and many new analogues and derivatives of anthracyclines antibiotics have been formulated for clinically trials [1–3]. Clinically proven anthracycline antibiotics are extensively used against acute leukemia, malignant lymphomas as well as have been found active in solid tumors; particularly in the case of breast cancer [4]. Molecular structures of anthracyclines contain a planar aglycone ring coupled with an amino-sugar which exhibits a variety of biological effects [5–7].

DNA is one of the targets of clinically used anticancer drugs including anthracyclines. Experimental and theoretical studies reported on anthracyclines for their binding with DNA have shown the possibilities of reversible binding interactions i.e., intercalation between the DNA base pairs via planar anthracycline ring and interaction of amino-sugar moiety with the negatively charged phosphate groups in the DNA minor groove that could lead to change in the shape of the DNA double helix and hinders DNA replication and RNA transcription [8–13]. Despite of anthracyclines being potent anticancer therapeutic agents, their clinical usefulness is limited due to their dose related cardiotoxicity [14].

Anthracycline induced cardiotoxicity have been associated with many factors which may include free radical formation in the heart, or from buildup of metabolic products (reactive oxygen species; ROS) of the anthracycline in the heart [15–17]. These ROS play an important role in DNA damage which is tightly related to mutagenesis, carcinogenesis, autoimmune inflammatory diseases, neuro-degenerative diseases and so on. Among ROS, hydroxyl radical (OH^.^) can attack all the molecules including DNA and play a major role in the formation of DNA oxidative damage. Therefore these drugs are taken in combination with the other drugs to reduce the side effects and reverse multi drug resistance [18, 19].

Antioxidants are considered powerful supplements and recommended to patients during cancer treatment. Investigation on various aspects of antioxidants used during cancer therapy is a prime subject in cancer research [20]. However free-radical formation by anthracycline is one of the major side effects of anthracyclines when used as chemotherapeutic drugs due to anthracycline-related lipid peroxidation [17]. Ascorbic acid being powerful antioxidant reduces anthracyclines to deoxyaglycons via a one-electron transfer mechanism; hence play an important role in increasing its drug efficiency by scavenging free radicals and decreasing cardiovascular toxicity [21, 22, 23].

Though antioxidants have been extensively used for their potentiality to prevent cancer in humans [24]; however, till yet there had been no studies on the therapeutic intervention of ascorbic acid with chemotherapeutic agents on molecular and cellular level. Present study is first time reported on electrochemical, spectroscopic and computational behavior of three anthracyclines—doxorubicin (DXH), epirubicin (EpiDXH) and daunorubicin (DNR) with DNA in the absence and presence of ascorbic acid under physiological conditions of pH and temperature. Some validations on cellular level are also carried out using non-small cell cancer cell lines H-157 and H-1299. Current study is aimed at role of ascorbic acid in increasing binding strength of anthracyclines with DNA and enhancing percent cell inhibition of anthracyclines in the presence of ascorbic acid.

## 2. Results and discussion

### 2.1 Cyclic voltammetry of anthracyclines and ascorbic acid

On bare glassy carbon electrode and in Mcillvaine buffer solution at pH 7.4 and 4.7; cyclic voltammetric responses and related electrochemical parameters showed one step reversible reduction process in DXH, while with EpiDXH and DNR reduction and oxidation processes, respectively, were observed with quasi behavior. While scanning the voltammograms, reduction peaks for DXH and EpiDXH were appeared in the forward scan at more negative potentials and oxidation peaks were appeared in the reverse scan at less negative potentials. Conversely, for DNR oxidation peak appeared at more positive potential in the forward scan, while reduction peak appeared at less positive potential when the scan was made reverse. Cyclic Voltammetry of AA showed irreversible oxidation at 0.66V. ΔE_p_, E_p_-E_p/2_ and current ratio (*i*_*p*_^*a*^*/i*_*p*_^*c*^) values of all compounds further confirmed the nature of electrochemical reaction. Voltammetric behavior of anthracyclines and ascorbic acid at PH 4.7 are given as S1–S3 Figs. (as peak-1; coloured blue) and electrochemical parameters at pH 7.4 and 4.7 are tabulated in S1 and S2 Tables provided in supplementary material.

### 2.2 Cyclic voltammetry of anthracycline–DNA and AA–DNA interactions

**Fig 1:**
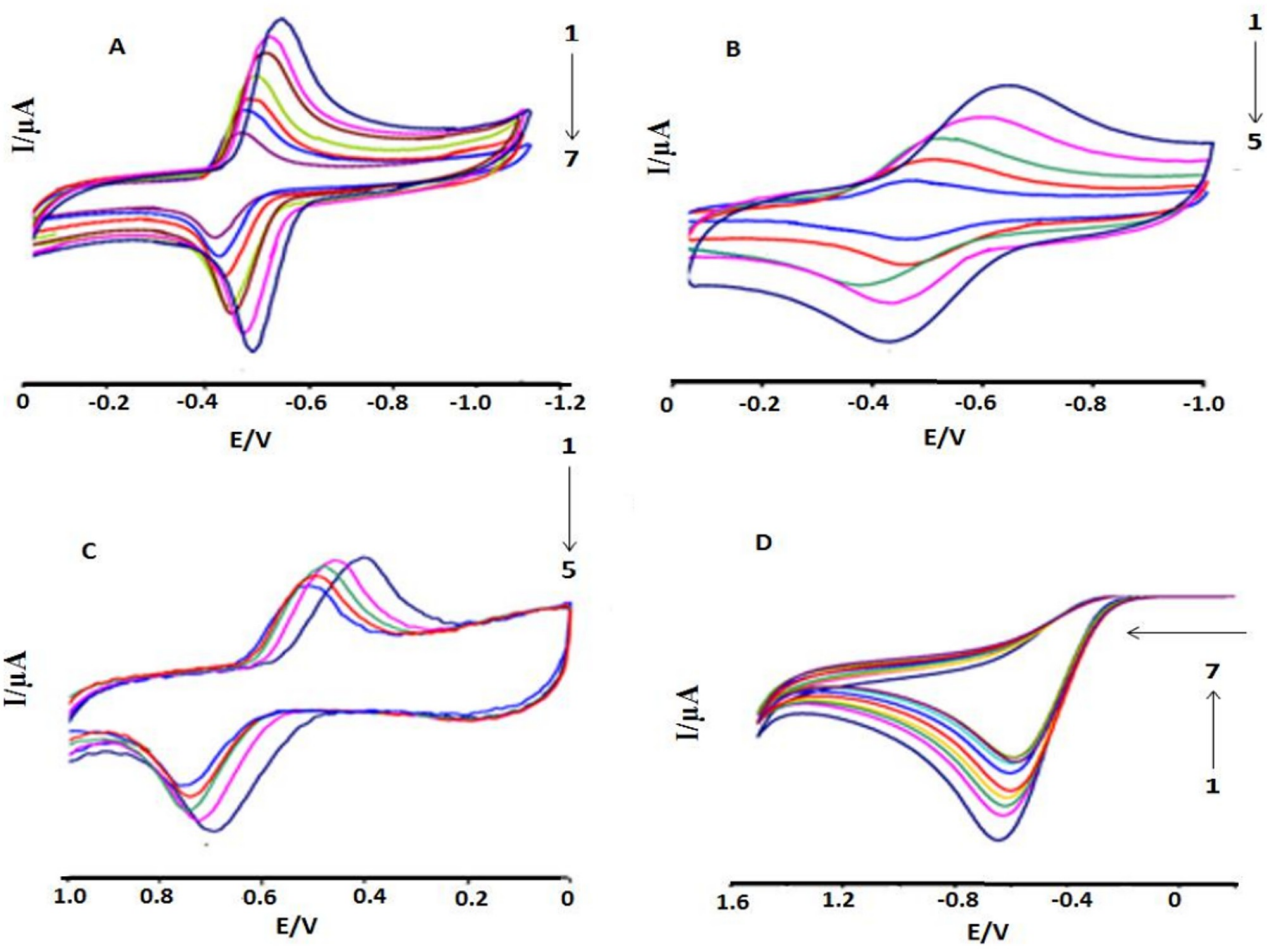
CV behavior of the interaction in Mcllvaine buffer between DNA and 5.0μM of (A) DXH (B) Epi-DXH (C) DNR and (D) AA. C_DNA_ = 0, 2μM, 2.5μM, 3μM, 3.5μM, 4.0μM, 4.5 μM; Arrow direction indicated the increasing concentrations of DNA, pH = 7.4, T = 309.5K.

CV behavior of anthracyclines and ascorbic acid were investigated separately in the presence of ds.DNA and shown in Fig 1 at pH 7.4,. DXH and Epi-DXH were reduced while DNR and AA were oxidized, when treated individually. Upon addition of various DNA concentrations (2–4.5μM), both oxidation and reduction peaks of anthracyclines gradually diminished in size. This decrease in current for both oxidation and reduction processes in a compound could be related to less availability of free anthracycline as interacted with DNA and that anthracycline–DNA adduct was formed. This decrease was observed maximum upon addition of DNA upto 4.5 μM in DXH and 3.5 μM in Epi-DXH and DNR and further additions resulted no change in peak current and peak potential.

In the presence of DNA, a considerable and gradual decrease in peak currents due to decrease in the equilibrium concentrations of anthracyclines was a typical evidence of drug–DNA interaction and was found in good agreement with the reported literature [9]. Further, anodic shifts (positive shifts in peak potentials) were observed in the CV responses of all anthracyclines upon successive addition of DNA concentrations and could be attributed to binding via hydrophobic interactions i.e., intercalation of compound into the bulky, slowly diffusing ds.DNA as reported by Bard and co-workers [25].

In case of ascorbic acid, a gradual decrease in the oxidation peak current with a slight negative shift (less positive potential) was observed after DNA addition upto 4.5 μM and such changes could be assigned as electrostatic interactions [25]. Voltammetric observations as well as E_p_-E_p/2_ and E_1/2_ values predicted the nature of the electrochemical reaction either reversible, quasi reversible or irreversible and the values showed that there is no change in the reaction nature for all anthracyclines and ascorbic acid before and after DNA addition.

### 2.3. Cyclic voltammetry of anthracyclines’ interactions with DNA and AA

It is known that AA has no effect on the antitumour activity of anthracyclines, but it significantly prolonged the life of patient treated with anthracyclines. These drugs elevate the lipid peroxide in heart muscles which is responsible for cardiotoxicity. AA prevents lipid peroxidation caused by these anthracyclines [26–28]. In present work, we made an attempt to study electrochemical interactions between anthracyclines with DNA in the presence of ascorbic acid by adopting two different routes. Complete electrochemical studies of these two routes are given in the following sections.

#### 2.3.1. Interaction of DNA with anthracycline–AA adduct: Route—1

**Fig 2:**
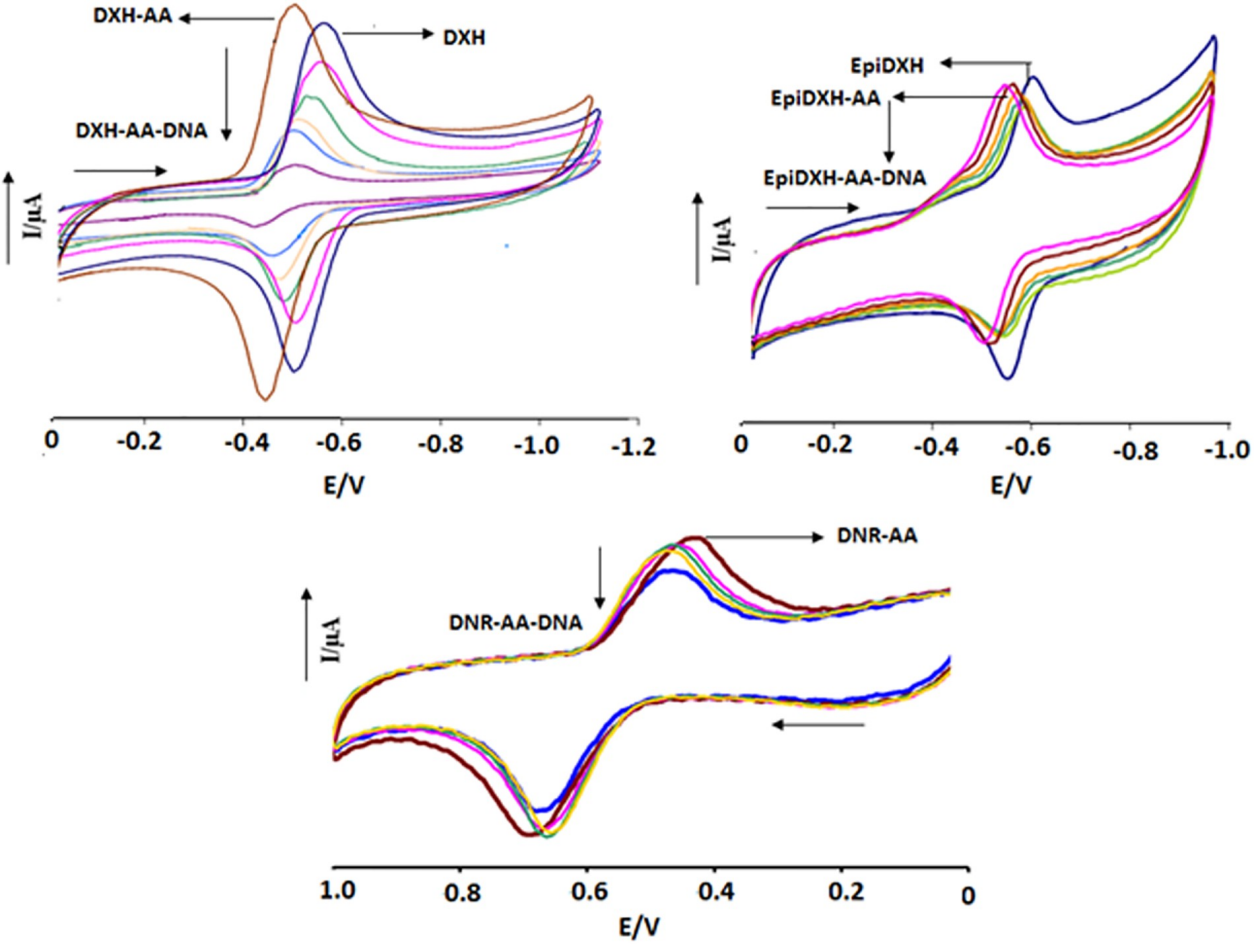
CV behavior of anthracyclines–AA adducts in the absence and presence of varying DNA concentrations. Arrow direction indicated the increasing concentrations of DNA, pH = 7.4, T = 309.5K.

In order to adopt Route–1; electrochemical interactions of anthracyclines with ascorbic acid were investigated by cyclic voltammetry. Significant anodic shifts in both cathodic and anodic peak positions were observed after the addition of 2 mM AA in 5 μM each of DXH, EpiDXH and DNR at both pH, Fig 2 at pH 7.4). Electrochemical response and current/ potential data predicted the possibility of adduct formation by interactions between anthracyclines and AA.

Via adopting Route–1, DNA was added in aliquots separately into the solutions of all the anthracyclines containing AA. Changes in the redox activity of all compounds were observed as decrease in the peak current heights with anodic shifts in peak potentials which revealed interaction of anthracycline–AA adduct with DNA, Fig 2 at pH 7.4. However, this anodic shift was found less pronounced as compared to that resulted due to anthracycline–AA adduct formation. Furthermore, similar to DXH and EpiDXH, E_1/2_ values for the DXH–A and EpiDXH–AA decreased as we increased DNA concentration which revealed ease in the reduction process. In comparison to DXH and EpiDXH adducts with AA, anodic shifts in peaks position were observed less pronounced for DNR–AA which showed less interaction of DNR with AA.

In the presence of various DNA concentrations, the redox potential of DNR–AA adduct showed no further shifts which is indicative of comparatively more stable nature of DNR–AA than DXH–AA and EpiDXH–AA, hence may not be decomposed when interacted with DNA. On the other hand, peak shifting in DXH–AA and EpiDXH–AA after the addition of DNA concentrations showed adduct instability due to which AA may be replaced by DNA hence forming an intercalated DXH–DNA and EpiDXH–DNA complexes. E_p_-E_p/2_ values evaluated for Route–1 complexes showed no change in the reaction reversibility as observed for compound–DNA adducts without ascorbic acid. At both pH (7.4 and 4.7) interactional behaviors was observed similar.

#### 2.3.2. Interaction of AA with anthracycline–DNA adduct: Route—2

**Fig 3:**
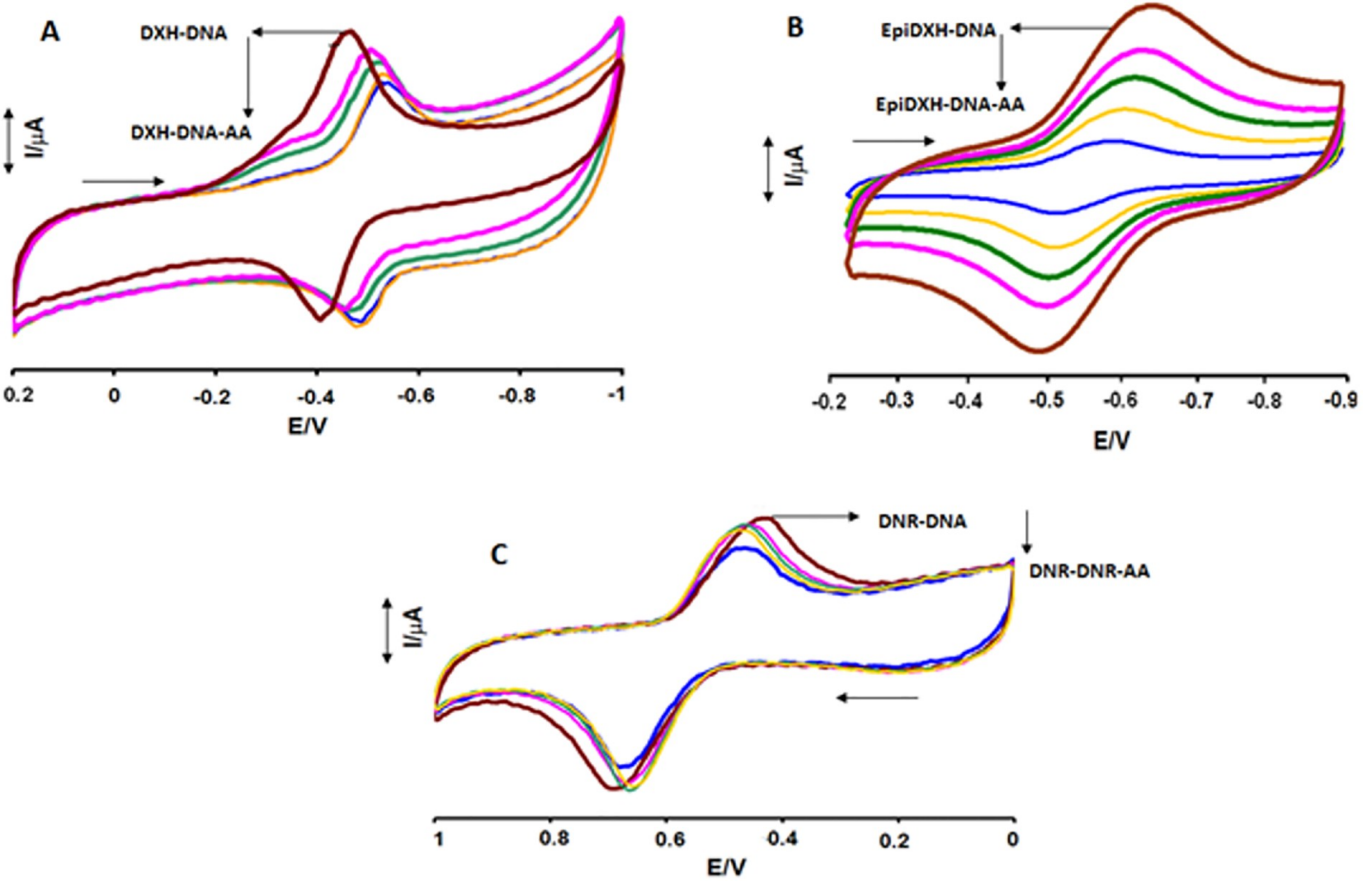
CV behavior of anthracyclines—DNA adducts in the absence and presence of varying AA concentrations. Arrow direction indicated the increasing concentrations of DNA, pH = 7.4, T = 309.5K.

In Route–2, Fig 3 at pH 7.4, changes in the electrochemical behavior of anthracycline–DNA adduct was investigated in the presence of different concentrations of AA. Anthracycline–AA adducts have shown substantial anodic shifts and current drops (both in *i*_*p*_^*a*^ and *i*_*p*_^*c*^*)* upon DNA additions in Route–1. However, in Route–2, when AA was added to all anthracycline–DNA adducts separately, both cathodic and anodic peaks were shifted towards less positive potential and reached nearly to oxidation and reduction potentials of DXH, EpiDXH and DNR. This behavior in peak shifting may be due to the replacement of DNA in anthracycline–DNA complex by AA. Furthermore, gradual decrease in the *i*_*p*_^*a*^ and *i*_*p*_^*c*^ currents of anthracycline–DNA adducts after the addition of increasing concentrations of AA provided evidences of AA interaction with DXH–DNA, EpiDXH–DNA and DNR–DNA adducts.

#### 2.3.3. Kinetic studies

Diffusion coefficients (*D*_*o*_) of DXH, EpiDXH and DNR adducts with DNA and AA have been evaluated by using Randles Sevcik equations {for reversible reaction; $i_{p}=2.69\times 10^{5}n^{3/2}AC_{o}^{*}D_{o}^{1/2}v^{1/2}$, for irreversible reaction; $i_{p}=2.99\times 10^{5}n{\left(\alpha n_{\alpha }\right)}^{1/2}AC_{o}^{*}D_{o}^{1/2}v^{1/2}$} [29, 30]. Plotting *i*_*p*_ values vs. υ^1/2^ showed linear dependency of peak current on square root of scan rate. The values of diffusion coefficients at both pH are provided in Table 1.

If we go into the morphology of the complexes formed with DNA in the presence of ascorbic acid (AA), their interpretation becomes quite complex. *D*_*o*_ values decreased insignificantly for all binary anthracycline–AA complexes when compared to simple anthracyclines. The minimal difference in the *D*_*o*_ values of compounds and their binary adducts with AA could be related to small size of AA, hence making no significant change in the size of complex. Whereas, complexes of DXH, EpiDXH and DNR with AA and DNA were found to have 10 times smaller *D*_*o*_ at both pH values due to the formation of large size tertiary complexes. The reduction in *D*_*o*_ values were evaluated 60.12%, 21.89% and 12.5% respectively for tertiary complexes of DXH, EpiDXH and DNR at pH 7.4. However, *D*_*o*_ values for AA–DNA complex were evaluated 10^2^ order of magnitude lesser than that calculated for AA alone at both pH. This larger drop in the *D*_*o*_ values could be attributed to the possibility that larger concentration of AA has interacted with DNA; hence reducing the diffusion ability of equilibrium mixture of AA–DNA complex to this extent (Table 1).

Effect of AA on the binding of anthracyclines with DNA in terms of heterogeneous electron transfer rate constant (*k*_*s*,*h*_) was also determined by using Kochi’s method, Eq 1(a), [31]. However, due to irreversible electrochemical behavior of AA heterogeneous electron transfer rate constants for AA and AA–DNA were calculated using Gileadi’s method, Eq 1(b), [32].

$$k_{s,h}=2.18{\left[\frac{D_{o}\alpha nF\upsilon }{RT}\right]}^{1/2}\text{exp}\;\left[-\frac{\alpha^{2}nF}{RT}\left(E^{a}{}_{p}-E^{c}{}_{p}\right)\right]$$ (1a)

$$\text{log}\;k_{s,h}=-0.48\alpha +0.52+\text{log}{\left[nF\alpha v_{c}D_{o}/2.303RT\right]}^{1/2}$$ (1b)

Where ‘*n’* is number of electrons transferred in redox process, *‘F’* is faraday’s constant, *ν* is the scan rate in V/s and α is transfer coefficient. *k*_*s*,*h*_ values for all the compounds and their binary tertiary complexes are provides in Table 1.

Decrease in *k*_*s*,*h*_ value*s* of DXH after adduct formation with AA were evaluated 64% and 25% respectively at pH 7.4 and 4.7 which showed that kinetic facility of DXH has decreased more significantly at pH 7.4 than that at pH 4.7. For tertiary complex DXH–AA–DNA, further decrease in *k*_*s*,*h*_ values revealed slow diffusion kinetic due to large sized tertiary complex. This decrease is more prominent at pH 7.4. Similar behavior was observed for binary and tertiary complexes of EpiDXH and DNR.

**Table 1:** Diffusion coefficients and heterogeneous electron transfer rate constants.

*D*_*o*_ (cm^2^ /s)			*k*_*s*,*h*_ × 10^3^ /cm/s	
Complexes	pH 7.4	pH4.7	pH 7.4	pH4.7
AA	9.33×10^−4 (oxidation peak)^	7.41×10^−4 (oxidation peak)^	1.19	2.33
DXH	3.66×10^−6^	2.86×10^−7^	42.0	10.06
EpiDXH	8.77×10^−6^	8.77×10^−6^	4.17	4.17
DNR	7.95 ×10^−6^	3.43 ×10^−6^	14.0	8.34
Binary complexes of DNA				
DXH-DNA	1.82×10^−6^	1.33×10^−8^	2.13	2.51
EpiDXH-DNA	6.95×10^−6^	8.19×10^−6^	1.25	1.29
DNR-DNA	5.48×10^−6^	4.33×10^−6^	2.71	5.93
AA-DNA	1.11 ×10^−6^	2.12 ×10^−6^	6.24	6.12
Binary complexes of AA				
DXH-AA	3.41×10^−6^	1.55×10^−7^	27.0	2.56
Epi DXH-AA	7.04×10^−6^	7.14×10^−6^	1.75	4.47
DNR-AA	6.43×10^−6^	2.75×10^−6^	5.71	6.43
Tertiary Complexes				
DXH-AA-DNA	2.20×10^−7^	1.12×10^−8^	2.09	1.77
EpiDXH-AA-DNA	1.92×10^−6^	2.21×10^−6^	1.95	4.14
DNR-AA-DNA	1.00×10^−6^	5.01×10^−7^	2.23	1.58

### 2.4. Electronic absorption spectroscopic studies of anthracyclines

**Fig 4:**
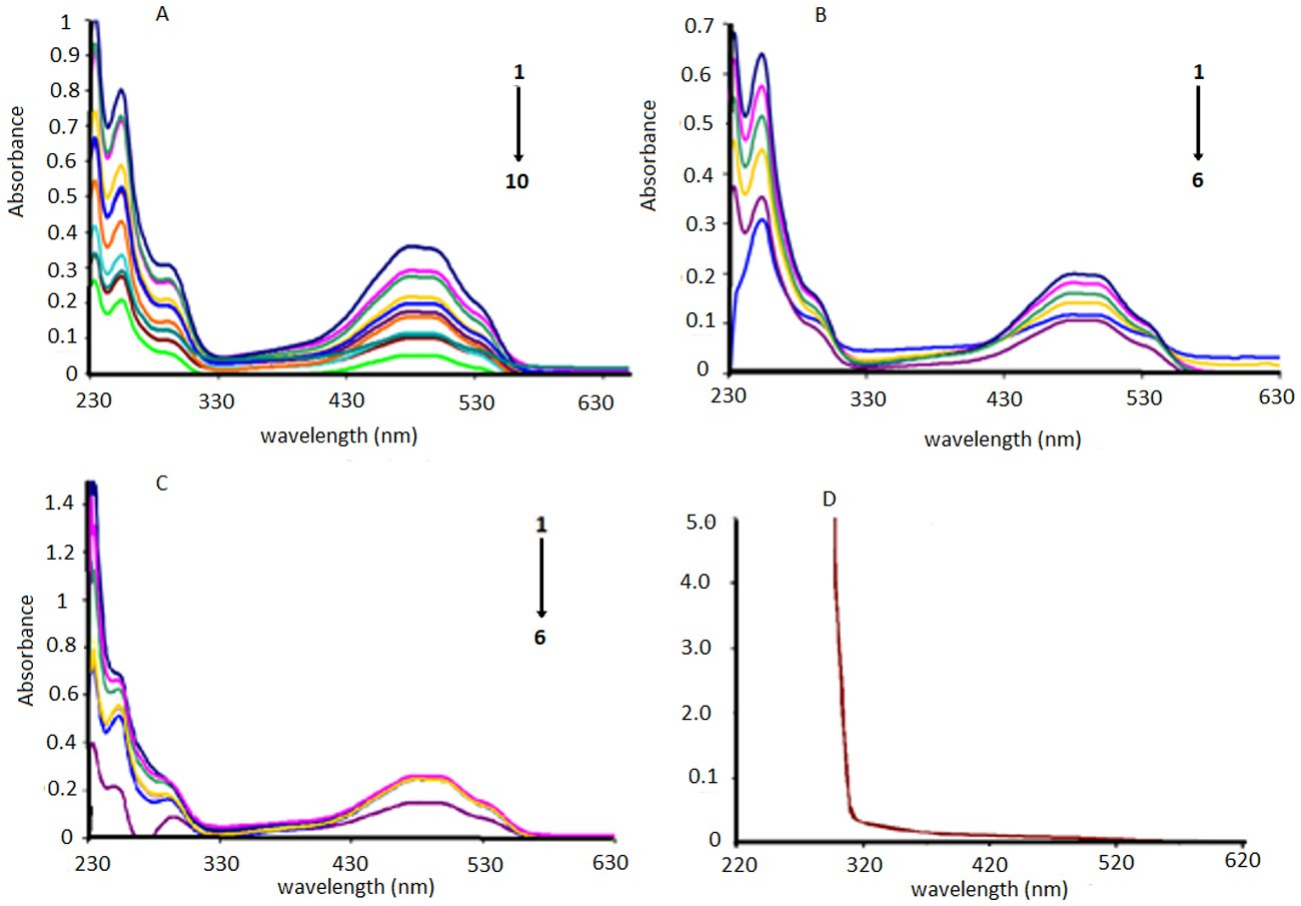
UV-Vis signature of the interaction in Mcllvaine buffer between DNA and 5.0μM of (A) DXH (B) Epi-DXH (C) DNR and (D) AA. Arrow direction indicated the increasing concentrations of DNA.C_DNA_ = 0, 2μM, 2.5μM, 3μM, 3.5μM, 4.0μM, 4.5 μM; 5.0 μM 5.5 μM 6.0 μM, pH = 7.4, T = 309.5K.

DNA–anthracycline complexes in the absence and presence of AA were further explored by electronic absorption spectroscopic experiments and their spectra are given in Fig 4 for pH 7.4, S4 Fig. at pH 4.7. Both DXH and EpiDXH have shown absorption maxima at the same wavelength (254 nm), while for DNR it appeared at 292 nm. One broader peak has also been observed at the higher wavelength region range (481nm) for all anthracyclines. Whereas within the spectral range of 200–800 nm; AA was found to be inactive, Fig 4. These absorptions in DXH, EpiDXH and DNR may be attributed to the electronic excitation from non-bonding p-orbital localized on oxygen atom to a π antibonding molecular orbital *i*.*e*; n→π* and π→π* transitions. Addition of different concentrations of DNA resulted in hypochromism due to intercalation of planar aromatic structural part of the DXH, EpiDXH and DNR. The electronic clouds of the planar anthracycline moiety interacted strongly with the electronic clouds of the DNA bases triggering hypochromic effect with no significant shift in peak position.

**Fig 5:**
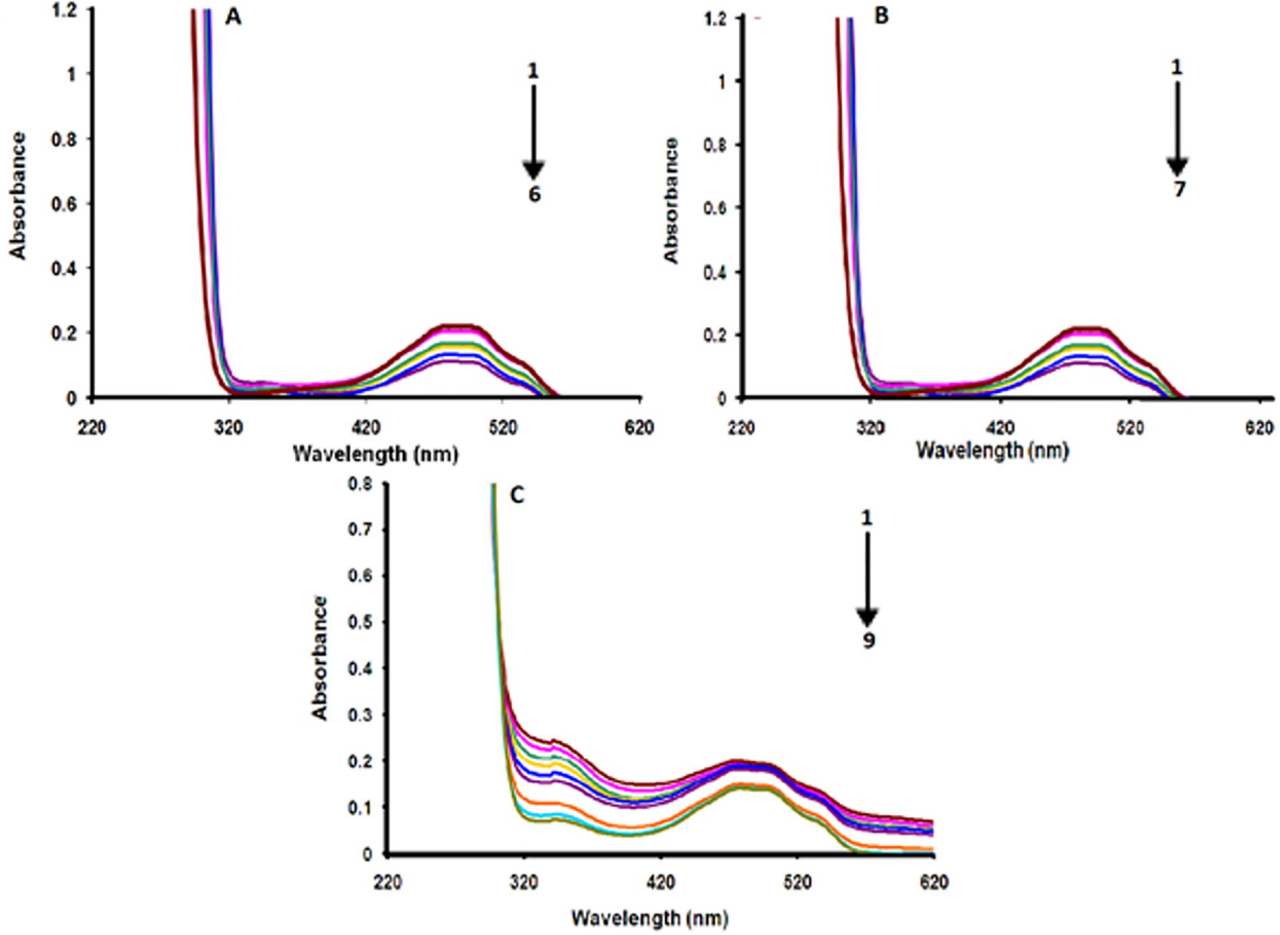
UV-Vis signatures of (A) AA–DXH, (B) AA–EpiDXH and (C) AA–DNR adducts in the absence and presence of varying DNA concentrations. Arrow direction indicated the increasing concentrations of DNA, pH = 7.4, T = 309.5K.C_DNA_ = 0μM, 2μM, 2.5μM, 3μM, 3.5μM, 4.0μM, 4.5μM, pH = 7.4, T = 309.5K.

Adopting Route–1, Fig 5, by pouring 2mM AA concentration to the DXH, EpiDXH and DNR, the peaks at 254nm and 292nm disappeared which showed that electronic transitions were restricted owing to the formation of hydrogen bonds between AA and hydoquinon and quinone moiety of DXH, EpiDXH and DNR. Upon addition of varying concentrations of DNA to the anthracycline–AA solution, absorbance peak at 481 nm decreased gradually which could be rationalized on the basis of a fact that anthracycline–AA adduct has been intercalated between the flanking DNA base pairs.

**Fig 6:**
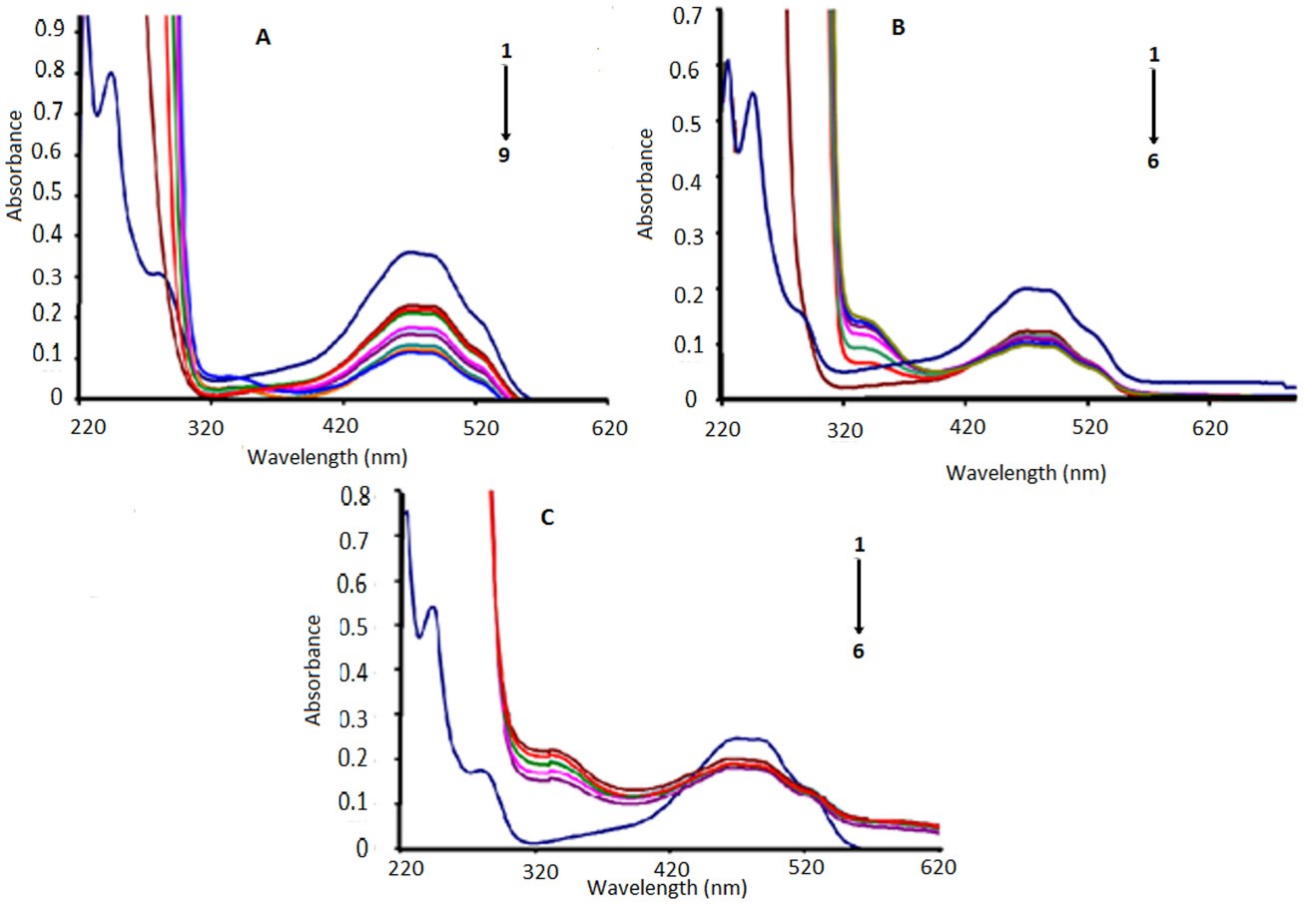
UV-Vis signatures of anthracyclines–DNA adducts in the absence and presence of varying AA concentrations. Arrow direction indicated the increasing concentrations of AA, pH = 7.4, T = 309.5K.

Similarly by adopting Route–2, Fig 6, by adding DNA to 5μM solution of DXH, EpiDXH and DNR, absorbance peak diminished without noteworthy peak shift. After the addition of different concentrations of AA into anthracycline–DNA solution, absorbance peaks at 292nm and 254 nm almost disappeared that may be happened due to restricted electronic transitions in these regions. Whereas, gradual decrease in absorbance peak at 481nm was observed.

### 2.5. Structural analysis by PM3

**Fig 7:**
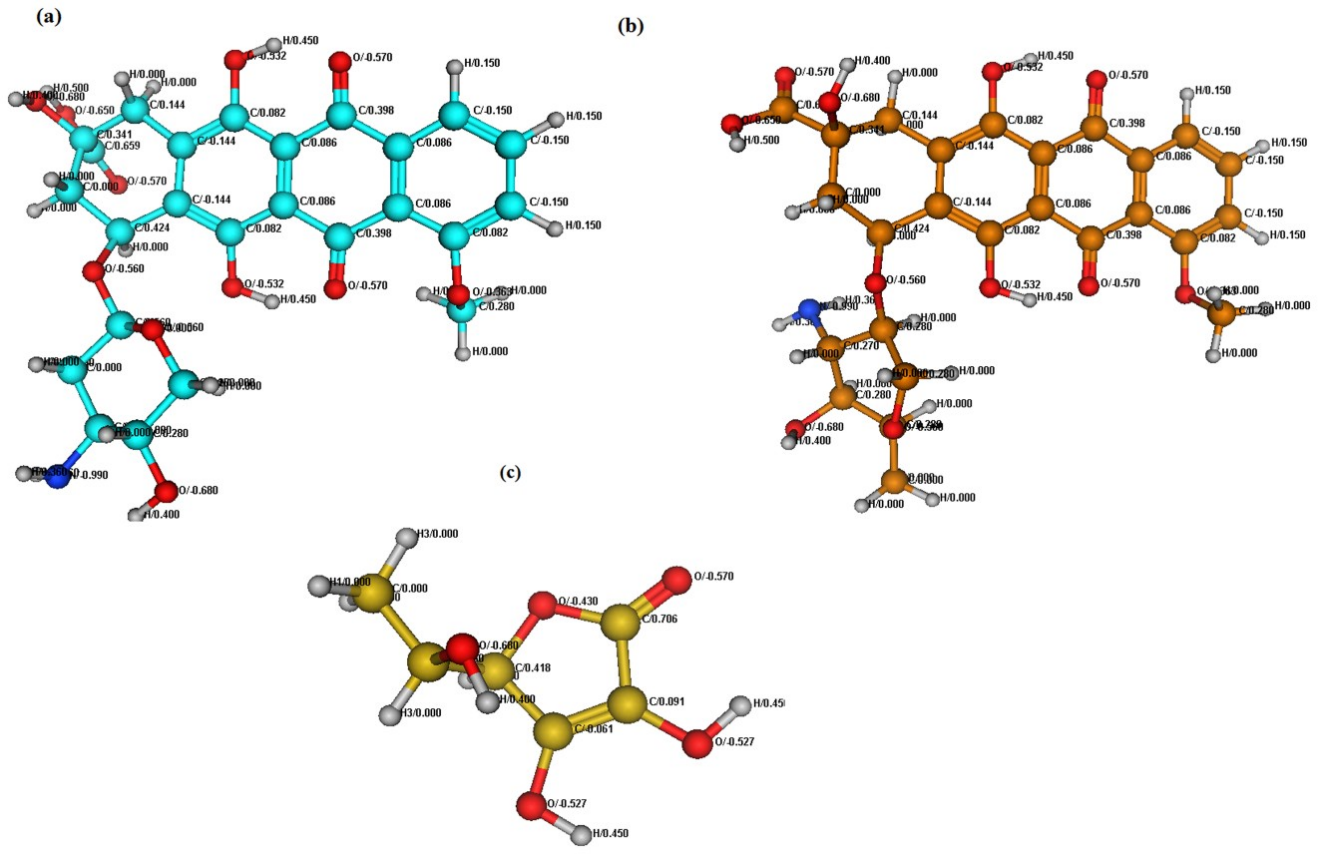
Optimized structures of DXH (a), DNR (b) and AA (c) using Semi-empirical PM3 method.

Semi-empirical PM3 method was used for structural elucidation of DXH, DNR and AA. Since EpiDXH is conformational isomer of DXH, therefore its theoretical analysis offered the results matching to DXH. Optimized structures and molecular charge distribution of DXH, DNR and AA are presented in Fig 7. Their structural exploration revealed highest electronegative atom in DXH, DNR and AA to be O atom with highest charge distribution of -0.680 whereas C atoms of planar aromatic ring are acting as highly electropositive thus intercalating between electron ring rich DNA base pairs Fig 7.

### 2.6. Molecular docking investigations

**Fig 8:**
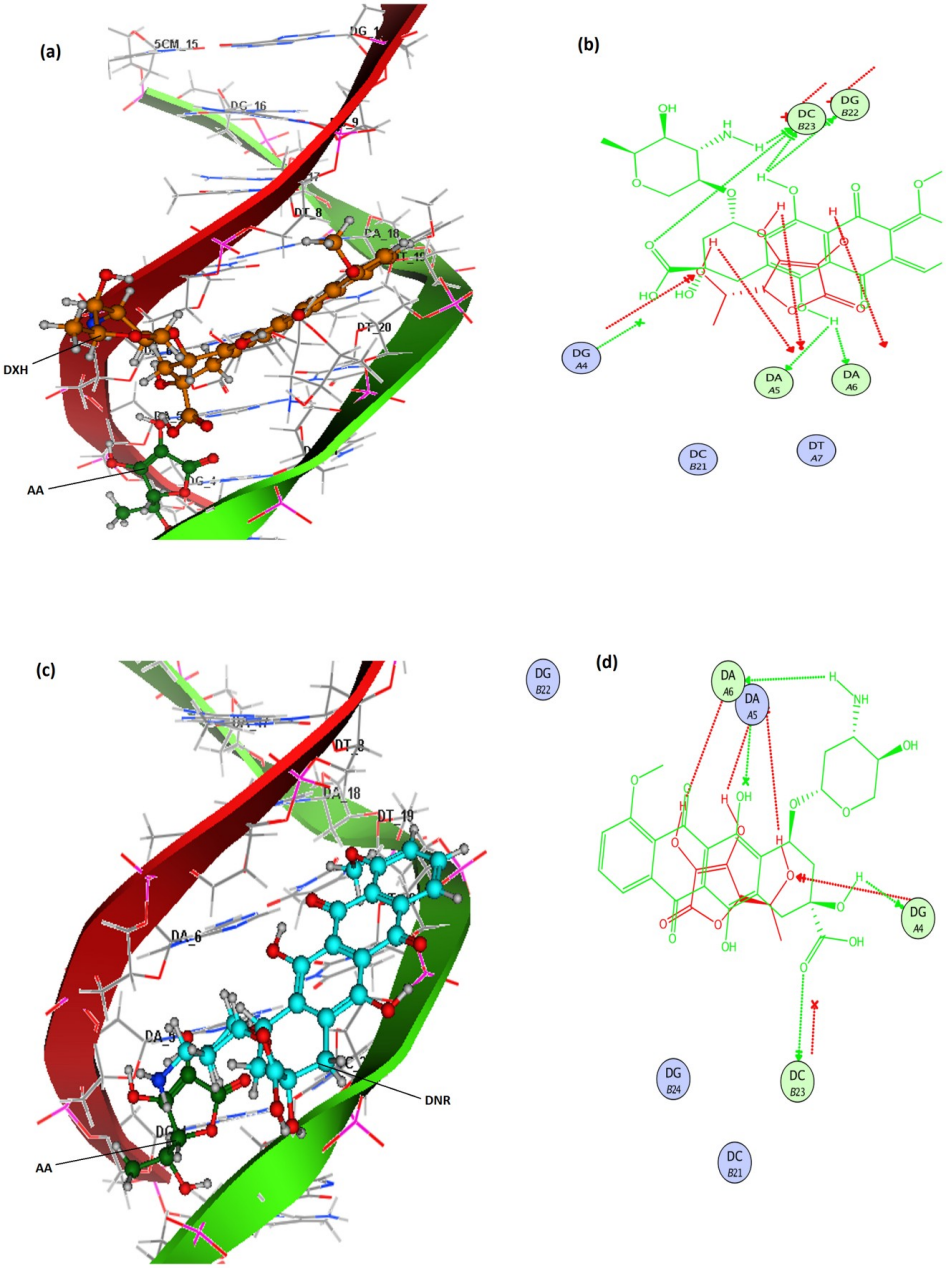
Tertiary docked complex of DXH (orange) and AA (green) with 1R2L(a); 2D-Ligplot showing potential binding interactions of DXH and AA with 1R2L base pairs (b);Tertiary docked complex of DNR (blue) and AA (green) with 1R2L (c); 2D-Ligplot showing potential binding interactions of DNR and AA with 1R2L base pairs (d).

Molecular docking studies of DXH and DNR were carried out in the presence of AA and shown in Fig 8. Since EpiDXH is stereoisomer of DXH, it has conformational poses for interaction with DNA was similar to that of DXH. Molecular docking analysis revealed increased interactions of DXH and DNR with DNA in the presence of AA. This could be related to the ability of AA to develop hydrogen bonding with DXH and DNR on one hand and intra strand cross linking with DNA on the other hand; hence increasing the strength of interaction and *K*_*b*_ values. 2D-lig plot of tertiary docked complexes of DXH and DNR with AA and DNA represented the nature of bonding with DNA base pairs. Fig 8(b) is illustrating that O atom in–COOH group of DXH has built hydrogen bonding with H- atoms of cytosine DC (B23), whereas H- atoms in hydroquinone group of DXH have developed hydrogen bonding with N atom of guanine DG (B22) on one strand and N of adenine DA (A5) on other strand. Additionally, oxygen atoms on AA further setup hydrogen bonding with H-atoms of adenine DA (A5) and DA (A6), thus multiplying strength of interaction.

Fig 8(d) determines 2D lig plot interaction profile of DNR with DNA in the presence of AA in terms of hydrogen bonding and hydrophobic interactions. In Fig 8(d), it can be observed that H-atom of–NH_2_ interacted with adenine molecules DA (A5) and DA (A6) via hydrogen bonding with N atom, –OH group of DNR has been attached with DG (A4) through hydrogen bond linkages with O atom of guanine, while oxygen atom of–COOH group in DNR was connected with–H atom of cytosine DC (B23). AA has enhanced all these interactions of DNR with DNA by connecting its H-atoms with nitrogen of A6 and A5 and O-atom with H- atom of DG (A4). Molecular Docking studies further showed enlargement of hydrophobic interaction sphere in the presence of AA; hence pharmacodynamics interactions of DXH, EpiDXH and DNR were increased synergistically.

### 2.7. Binding constants of drug–DNA complex

Binding constants (*K*_*b*_) and binding site size (s) of anthracycline complexes with DNA and AA were calculated from cyclic voltammetric, UV-visible spectroscopic and molecular docking data by using Eqs (2), (3) and (4), respectively [33– 35].

$$i_{p}^{2}=\frac{1}{K_{b}[DNA]}(i_{p}{}_{0}^{2}-i_{p}{}^{2})+i_{p}{}_{0}^{2}-[DNA]$$ (2)

$$\frac{A_{o}}{A-A_{o}}=\frac{\varepsilon_{G}}{\varepsilon_{H-G}-\varepsilon_{G}}+\frac{\varepsilon_{G}}{\varepsilon_{H-G}-\varepsilon_{G}}\frac{1}{K_{b}\left[DNA\right]}$$ (3)

$$\Delta G=-RT\;\text{ln}\;K_{b}$$ (4)

In Eq 2, i_p_ and i_po_ are the peak currents with and without DNA. A plot of i_p_^2^ vs. (i_po_^2^– i_p_^2^)/ [DNA] gave a straight line with a slope equal to the reciprocal of binding constant, *K*_*b*_. In Eq 3, A_o_ and A, E_G_ and E_H-G_ represents absorbance and molar extinction coefficients of a compound and its DNA bound complex, respectively. From the plot of A_o_/(A- A_o_) to 1/[DNA]; the ratio of the intercept to the slope gave the values of binding constant, *K*_*b*_. While, Δ*G* obtained from the docked results was substituted in Eq 3 to obtain the value of *K*_*b*_. The same Eq 3 was used to evaluate Δ*G* using experimentally calculated *K*_*b*_ from Eqs 2 and 3. Further, the binding site sizes were evaluated through cyclic voltammetry by using following equation [33];

$$i-i_{DNA}/i_{DNA}=K_{b}[\text{DNA}]/2\text{s}$$ (5)

Where, *i* and *i*_*DNA*_ are the peak currents of a compound in the absence and in the presence of DNA, respectively, and “s” is the binding site size in terms of base pairs (bp). Putting the value of *K*_*b*_ as calculated according to Eq (2), the binding site size was obtained from the plot of *i*–*i*_*DNA*_ / *i*_*DNA*_ = *K*_*b*_[DNA] / 2s.

The values for binary complexes with DNA and AA, and tertiary complexes following Route–1 and Route–2 are provided in Table 2 for pH 7.4, S3 Table for pH 4.7. The order in the *K*_*b*_ values for all the types was observed as follows;

$$K_{b\left(tertiary\;complex\text{-}Route\text{-}1\right)}>K_{b\left(tertiary\;complex\text{-}Route\text{-}2\right)}>K_{b(}{}_{\text{binary}\;\text{complexes}\;\text{with}\;\text{DNA})}>K_{b\;(}{}_{\text{binary}\;\text{complexes}\;\text{with}\;\text{AA})}$$

The same order was obtained for Δ*G*. The values for tertiary complexes from Route–1 were found significantly higher, while least values of binary complexes with AA showed weakest type of interactions. The greater *K*_*b*_ and Δ*G* obtained from Route–1 revealed strongest and more favorable interaction of AA–anthracycline complex with DNA base pairs via intercalation. Comparatively lower *K*_*b*_ and Δ*G* values obtained in Route–2 than in Route–1 may be attributed to replacement of some proportion of DXH, EpiDXH and DNR by AA; as AA was added on anthracycline–DNA complexes.

Δ*G* values for all the binary and tertiary complexes were evaluated negative which showed that all the binary and tertiary processes involved in complex formations were spontaneous. The binding site size values for the tertiary complexes obtained from Route–1 were evaluated greater than that obtained from Route–2, which further justified stronger interactions due to the availability of more binding sites. Among all the tertiary complexes from Route–1, greatest binding site size value (2.72) of DXH–AA–DNA complex showed comparatively stronger interaction than that for DNR–AA–DNA and EpiDXH–AA–DNA complexes at pH 7.4.

**Table 2:** Binding parameters obtained from experimental and theoretical studies at pH 7.4 and T = 309.5K.

Experimental						Theoretical	
Cyclic Voltammetric				UV-Vis spectroscopic		Molecular docking	
complexes	*K*_*b*_/M^1^×10^5^	-ΔG/kJmol^-1^	Binding site size (s)/bp	*K*_*b*_/M^-1^×10^5^	-ΔG/kJmol^-1^	*K*_*b*_/M^-1^×10^5^	-ΔG/kJmol^-1^
Binary complexes							
AA–DNA	0.034	20.92	2.00	0.02	19.55	0.006	16.46
DXH–DNA	5.00	33.76	1.60	0.17	25.06	3.55	32.88
EpiDXH–DNA	0.11	23.94	1.31	0.14	24.56	0.43	27.45
DNR–DNA	17.2	36.94	1.51	11.80	63.90	42.81	39.29
Binary complexes							
DXH–AA	0.0076	17.06	----------	0.017	19.14	0.064	21.71
EpiDXH–AA	0.006	16.46	-----------	0.016	18.98	0.064	21.71
DNR–AA	0.00089	11.55	----------	0.028	20.42	0.047	20.94
Route–1 Tert. complexes							
DXH–AA–DNA	200.0	43.25	2.72	8.72	35.19	7.31	34.74
EpiDXH–AA–DNA	3.99	33.18	1.55	5.50	34.01	5.93	34.20
DNR–AA-DNA	31.91	38.53	2.00	23.23	31.40	24.61	37.86
Route–2 Tert. complexes							
DXH–DNA–AA	30.0	38.37	0.81	1.36	30.41	2.73	32.20
EpiDXH–DNA–AA	1.21	30.11	0.55	2.49	31.97	1.54	30.73
DNR–DNA–AA	2.31	31.779	1.00	0.23	25.84	4.61	33.55

### 2.8. Cell culture studies

**Fig 9:**
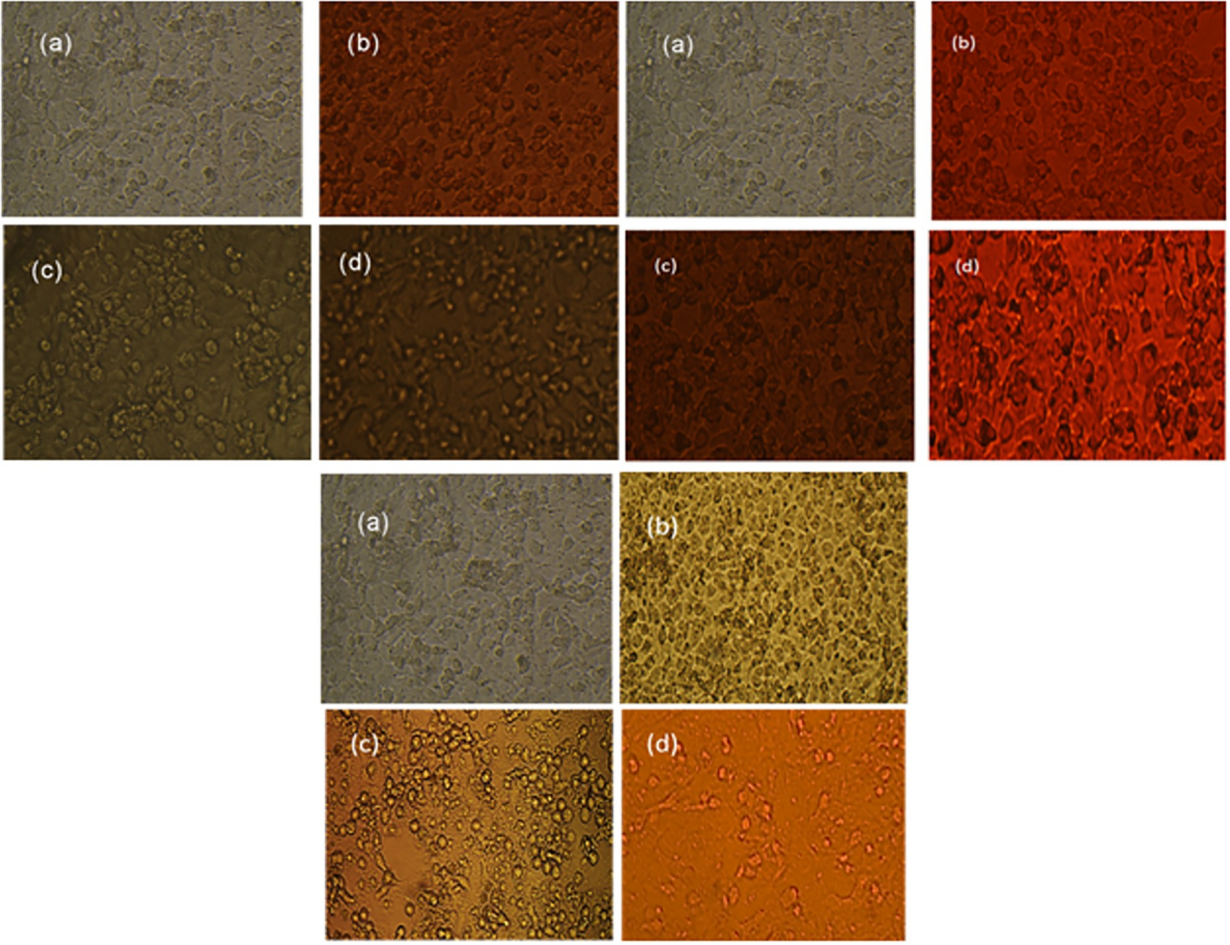
(a) Control for cell line H-157, (b) intracellular drug distribution (2mM drug) and drug-induced cell damage, (c) intracellular AA distribution (2mM AA) and AA induced cell damage, (d) intracellular drug distribution (IC_50_ coadministerd with 2mM AA) and drug-induced cell damage.

Proliferation of non-small cell cancer cell lines (NSCCLs) H-157 and H-1299 have shown inhibition by DXH, EpiDXH and DNR in the absence and presence of AA, Fig 9 for H-157 and S5 Fig for H-1299. Percent cell inhibition (%C_inh_) of H-157 and H-1299 by DXH, EpiDXH and DNR without and in the presence of AA were evaluated from Eq 6 and provided in Table 3 along with respective IC_50_ values [36].

%Cinh=100−{(At−A)b/(Ac−Ab)×100 (6)

**Fig 10:**
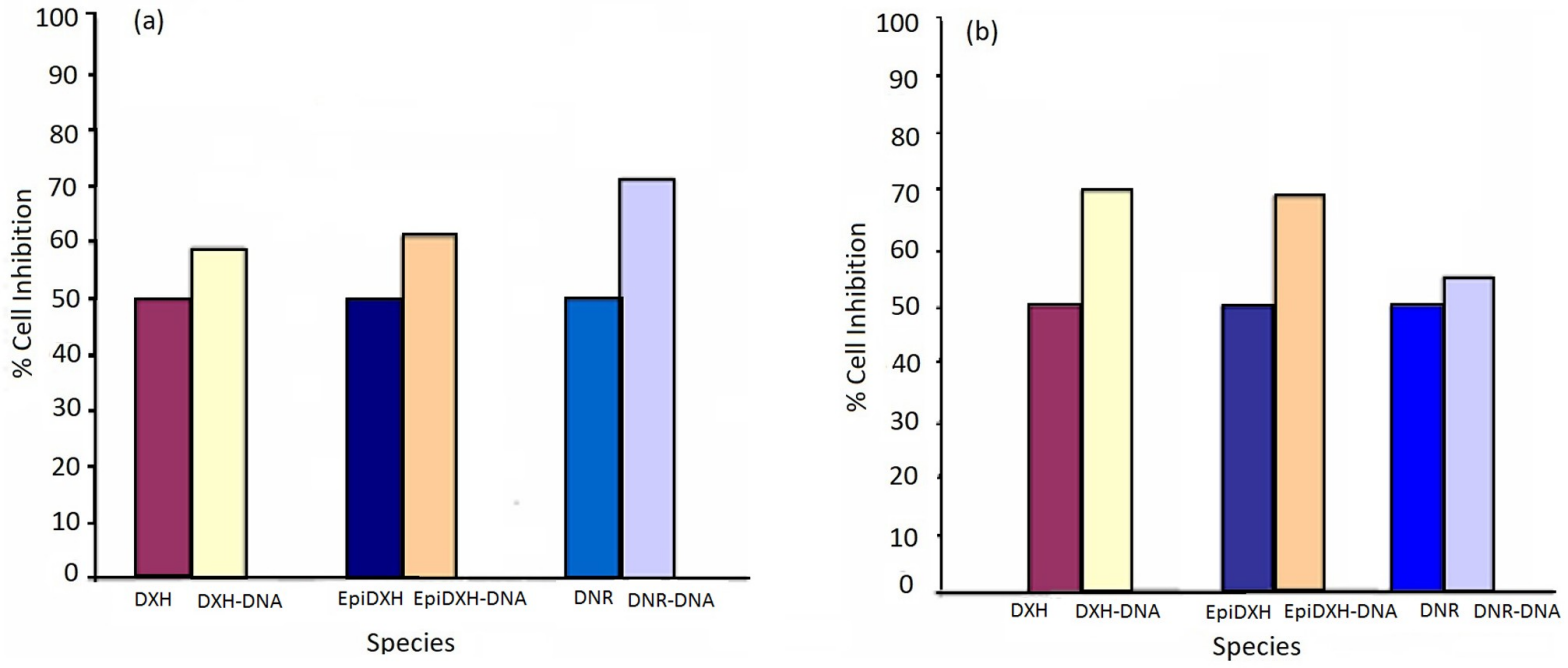
Percent cell inhibition of DXH, EpiDXH and DNR against (a) H-1299 and (b) H-157 cell lines.

Where, A_b_, A_c_ and A_t_ is absorbance value of blank, control and test compound respectively. For both human cancer cell lines H-157 and H-1299 percent cell inhibition (%C_inh_) was significantly enhanced in the presence of AA for each of DXH, EpiDXH and DNR as shown in Fig 10, thus reducing the IC_50_ values of the three anthracyclines. Minimum IC_50_ value was exhibited by DNR as it caused 50% cancer cells death at its minimum concentration of 310 μM and 330 μM respectively for H-157 and H-1299 cell lines. Further, highest *K*_*b*_ value determined experimentally and theoretically demonstrated highest binding strength of DNR, Table 3. DXH anticipated highest IC_50_ values for both NSCLCs cell lines. Percent cell inhibition of the anthracyclines with concentration corresponding to their IC_50_ values in the presence of 100μM, 500 μM, 1mM and 2mM of AA were also explored. Incorporation of low doses of AA (500 μM and 1mM) did not affect the cell inhibition of anthracyclines. However, addition of 2mM ascorbic acid has increased the %C_inh_; hence enhanced the efficiency of all three anthracyclines at this concentration, Fig 9.

**Table 3:** Percent cell inhibition (%C_inh_) and IC_50_ data.

Administered drugs	%C_inh_ of Drug (2mM)	%C_inh_ of Drug-AA	% increase in C_inh_	IC_50_^(H-1299)/^μM	IC_50_^(H-157)/^μM
DXH	57.88	83.94	45.04	500	410
EpiDXH	56.75	82.91	46.09	498	408
DNR	68.75	97.29	41.51	330	310

## 3. Conclusions

Interactional studies of three anthracyclines i.e., DXH, EpiDXH and DNR with DNA were carried out in the absence and presence of ascorbic acid (AA) by adopting two Routes. Results revealed that binding propensity of anthracyclines with DNA was comparatively high when AA was added to anthracycline before addition of DNA (Route–1; anthracycline–AA–DNA) than after DNA addition (Route–2; anthracycline–DNA–AA). Molecular docking also predicted greater interactions of drug with DNA in the presence of AA due to increased hydrogen bonding of anthrayclines. Pharmacodynamic interactions at cellular level validated that AA is anticipated to potentiate the investigated anthracyclines for the treatment of H-157 and H-1299 cancer cell lines by increasing the percent cell inhibition values. An important conclusion that was drawn from this work is binding interactions of anthracyclines with DNA that were increased in the presence of AA. It was further be concluded that the anthracycline drug dosage, that can cause greater DNA apoptosis, could be reduced in the presence of AA and may resulted in fewer toxic side effects of the drug if used alone.

## 4. Materials and methods

### 4.1. Reagents and chemicals

Clinical grade doxorubicin (DXH), epirubicin (EpiDXH) and daunorubicin(DNR) were obtained from Actavis, Italy, Pharmaceuticals and in view of the toxicity of DXH, EpiDXH and DNR, special care was paid. Ascorbic acid (AA) was purchased from Merck, Darmstadt. Disodium, while hydrogen phosphate, citric acid and EDTA of analytical purity grade were purchased from BDH. Tissue culture plates were purchased from Falcon, Oxnard, California, U.S.A. The reagents were applied without further purification.

### 4.2. Solution preparations

Double-stranded (ds) chicken blood DNA was extracted by falcon method. It was dissolved in doubly distilled water at a final concentration of 2.00 × 10^**−4**^ mol L^**−1**^ and stored at 0–4 °C. The concentration of the stock solution was determined by UV-absorbance at 260 nm using the molar extinction coefficient (ε) of 6600 M^**-1**^ cm^**-1**^. A ratio of absorbance at 260 nm to that at 280 nm, (A_**260**_/A_**280**_) greater than 1.8 indicated that DNA was sufficiently pure and free from protein [37]. 0.12M Mcillvaine buffer (0.2 M Na_2_HPO_4_.2H_2_O + 0.1M citric acid) was prepared and used as supporting electrolyte. Stock solutions of the drugs were prepared in distilled water and diluted with 0.12 M Mcillvain buffer immediately before use. Solution of ascorbic acid was also prepared in Mcillvain buffer. Doubly distilled water was used for all preparations.

### 4.3. Instrumentations

Voltammetric measurements were carried out using Autolab PGSTAT-302 (Echo Chemie, Utrecht, Netherlands) with software package GPES version 4.9. An electrochemical cell (Model K64 PARC) was used in CV studies that consisted of saturated calomel (Fischer Scientific; filling solution: saturated KCl (3.5M) solution), thin Pt wire (5 ×10^−4^ m thickness with an exposed end of 10^−2^ m) and glassy carbon (geometric area; 7.1 × 10^−6^ m^2^) as reference, counter and working electrode, respectively. The absorption was measured and corresponding spectra were obtained on a Shimadzu UV-1601PC UV–Vis scanning spectrophotometer. The pH was measured using pH meter Model M64 (ORION). Molecular docking was performed using MOE- Chemical computing Inc., 2015. Molecular structures of DXH, EpiDXH, DNR and AA were drawn and optimized on MOE window using MOE builder and moved into MOE database.

### 4.4. Experimental procedures for DNA binding analysis

#### 4.4.1. Cyclic voltammetry experiments

Sample solutions of drugs were maintained at pH 4.7 and 7.4 (human stomach and blood pH, respectively). For electrochemical analysis, sample solution was placed in an electrochemical cell and cell temperature was maintained at 36.5°C (human body temperature). Purging with nitrogen gas was made for 8 min before the commencement of the experiments and 2 min purging time was maintained between each measurement. Cyclic voltammograms of doxorubicin (DXH) and epirubicin (EpiDXH) were scanned within the potential rang of 0 –-1.2V, while for scanning daunorubicin (DNR) and ascorbic acid (AA) potential scan ranges were selected as +I– 0 V and +1.6 –-0.2V respectively. For binding studies, voltammetric responses were obtained after titrations of varying concentrations of DNA (2.0–4.5 μM) with constant drug concentration (5 μM) and then after adding different aliquots of ascorbic acid (1.2–2.8 mM) into drug–DNA solutions [38].

#### 4.4.2. UV-visible spectroscopy experiments

Absorption spectra of all investigated drugs were obtained before and after the addition of DNA and AA using UV-visible spectrophotometer. Similar titration procedure, under similar physiological conditions, was adopted for spectral analysis as done for electrochemical experiment. The aliquots of DNA and AA used were in the range 2.0–6.0 μM and 1.2–4.4 mM, respectively. The temperature of cell cuvette was maintained at 36.5°C by temperature controller device before running the instrument for the sample analysis [39].

### 4.5. Theoretical procedures for DNA binding analysis

#### 4.5.1. Semi-empirical PM3 method

Semi-empirical PM3 method was used for drawing structures of the anthracycline drugs and ascorbic acid. Geometries of the compounds were optimized and charges on reactive sites of each compound were computed [38].

#### 4.5.2. Molecular docking protocol

For the conformational analysis of drug binding with DNA, molecular docking was performed using MOE- Chemical computing Inc,. 2015. Molecular structures of DXH, EpiDXH, DNR and AA were drawn and optimized on MOE window using MOE builder and moved into MOE database. Three dimensional structure of DNA (PDB ID 1R2L) was downloaded from www.rcsb.org and its pdb.gz file was imported to MOE window and viewed on MOE window by opening its pdb.gz file. Before the docking process, the non-polar hydrogen atoms were added and water was removed from the structure by selecting water chains in MOE sequence editor. Standard geometry of 1R2L was optimized using MOPAC 7.0. The optimized structure was then subjected to systematic conformational analysis with RMS gradient of 0.01 kcal mol^-1^. For the purpose of finding putative docking pose, a number of conformational runs were carried out. Interaction energies of DXH, EpiDXH, DNR in the absence and presence of AA was evaluated from scoring function. The best docking conformation having minimum final docking energy or free energy (ΔG) was used for further calculations throughout. Binding propensity of drugs with DNA in terms of binding constants (*K*_*b*_) was calculated from the lowest ΔG values [38, 39, 40]. Remaining parameters were set default.

### 4.6. Cell culture assays

Exponentially growing cells of H-1299 and H-157 purchased from ATCC Number CRL-5803 and Sigma Aldrich were harvested with trypsin (0.05%): EDTA (0.02%) and re-suspended to a final concentration of 8.2 x l0^5^ cells/mL in fresh medium RPMI 1640. Cell suspensions (100 μL) were dispensed into the individual wells of a 96-well tissue culture plate. Each plate contained medium alone in one row and culture of cells in other row. Plates were re-incubated. After 24-h incubation, solutions of eight anticancer drugs (100 μL) at different concentrations (starting from higher to the lower concentration) were added to individual wells containing cells. The plates were then incubated under the same conditions for 24 h. Similar procedure was carried out for assay making in the presence of ascorbic acid. In order to investigate the combination effect of AA, % cell Inhibition of the drugs in the presence of 2mM AA was determined. At the end of incubation time, plates were removed from incubator after fixing with 50% TCA (trichloroacetic acid) and a process of staining followed by washing and solubilization in 10mM Tris buffer. Readings were collected using PLATOS496 [41].

## Supporting information

S1 Fig CV behavior of the interaction in Mcllvaine buffer between DNA and 5.0μM of (A) DXH (B) Epi-DXH (C) DNR and (D) AA. {C_DNA_ = 0, 2μM, 2.5μM, 3μM, 3.5μM, 4.0μM, 4.5 μM; arrow direction indicated the increasing concentrations of DNA, pH = 4.7, T = 309.5K}.
(PDF)

S2 Fig CV behavior of AA–anthracycline adducts in the absence and presence of varying DNA concentrations. Arrow direction indicated the increasing concentrations of DNA. pH = 4.7, T = 309.5K.
(PDF)

S3 Fig CV behavior of DNA–anthracycline adducts in the absence and presence of varying AA concentrations. Arrow direction indicated the increasing concentrations of DNA. pH = 4.7, T = 309.5K.
(PDF)

S4 Fig UV-Visible signatures for the interaction in Mcllvaine buffer between DNA and 5.0μM of (A) DXH (B) Epi-DXH (C) DNR and (D) AA. {C_DNA_ = 0, 2μM, 2.5μM, 3μM, 3.5μM, 4.0μM, 4.5 μM; 5.0 μM. Arrow direction indicated the increasing concentrations of DNA, pH = 4.7, T = 309.5K}.
(PDF)

S5 Fig (a) Control for cell line H-1299, (b) Intracellular drug distribution (2mM drug) and drug-induced cell damage, (c) Intracellular AA distribution (2mM AA) and AA induced cell damage, (d) Intracellular drug distribution (IC_50_coadministerd with 2mM AA) and drug-induced cell damage. (PDF)

S1 Table Electrochemical parameters of DXH, EPiDXH and DNR in the absence and presence of different concentrations of DNA at pH 7.4 and 4.7{buffer: 0.12M Mcllvainesolution,Temp: 309.5K, Scan rate:0.1V/s. (PDF)

S2 Table Electrochemical parameters of DXH–AA, EPiDEX–AA and DNR–AA in the absence and presence of different concentrations of DNA at pH 7.4 and 4.7. {buffer: 0.12M Mcllvaine solution, Temp: 309.5K, Scan rate: 0.1V/s.
(PDF)

S3 Table Binding parameters obtained from Cyclic Voltammetricand UV-Vis spectroscopic studies at pH 4.7and T = 309.5K. (PDF)
