# Supplementary material for: Electrochemical, spectroscopic and theoretical monitoring of anthracyclines’ interactions with DNA and ascorbic acid by adopting two routes: Cancer cell line studies
Source: PLoS One. 2018 Oct 29;13(10):e0205764. doi: 10.1371/journal.pone.0205764 (PMC6205586; doi:10.1371/journal.pone.0205764)
Supplement: S2 Fig — Arrow direction indicated the increasing concentrations of DNA. pH = 4.7, T = 309.5K. (PDF) [file pone.0205764.s002.pdf]

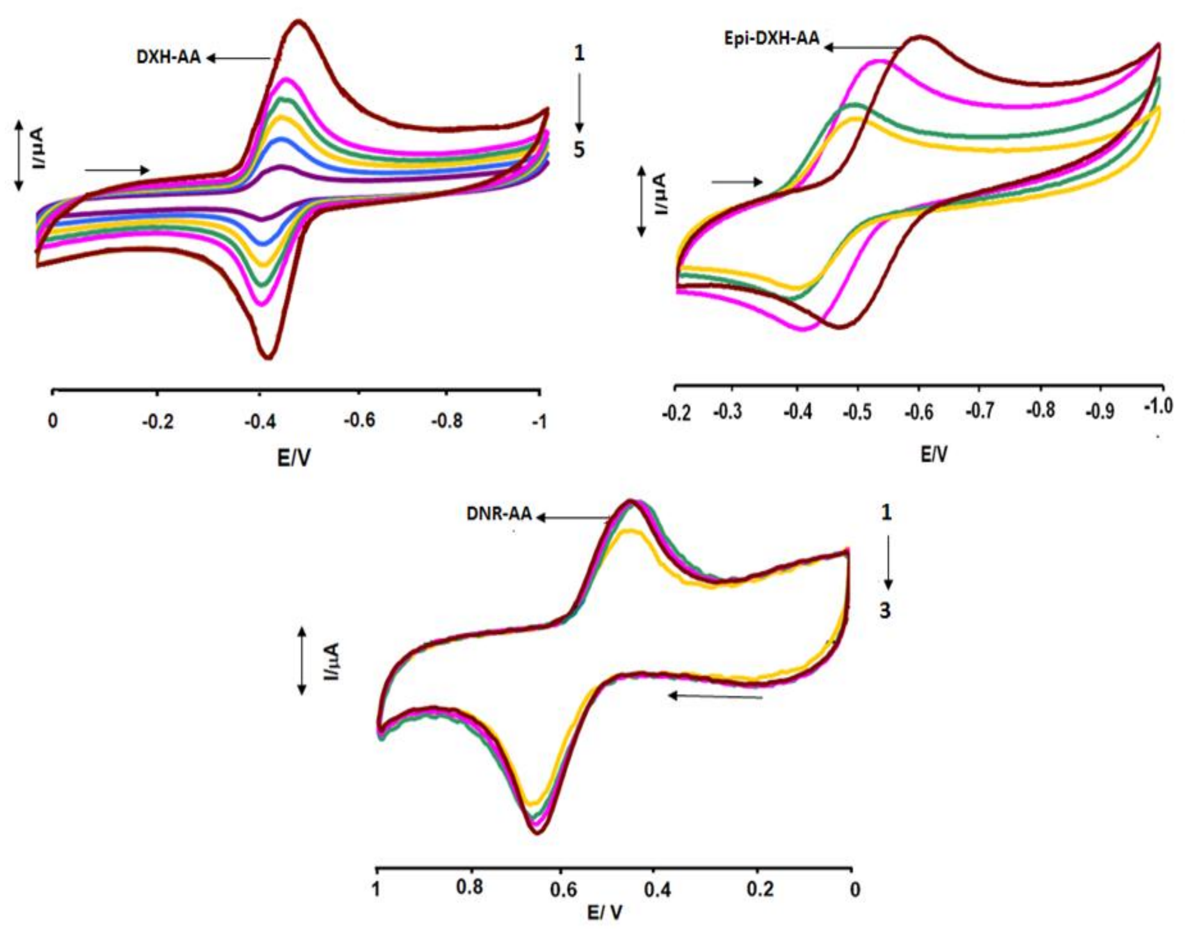

S2 Fig. CV behavior of AA–anthracycline adducts in the absence and presence of varying DNA concentrations. Arrow direction indicated the increasing concentrations of DNA. pH = 4.7, T=309.5K.
