# Supplementary material for: Electrochemical, spectroscopic and theoretical monitoring of anthracyclines’ interactions with DNA and ascorbic acid by adopting two routes: Cancer cell line studies
Source: PLoS One. 2018 Oct 29;13(10):e0205764. doi: 10.1371/journal.pone.0205764 (PMC6205586; doi:10.1371/journal.pone.0205764)
Supplement: S4 Fig — {CDNA = 0, 2μM, 2.5μM, 3μM, 3.5μM, 4.0μM, 4.5 μM; 5.0 μM. Arrow direction indicated the increasing concentrations of DNA, pH = 4.7, T = 309.5K}. (PDF) [file pone.0205764.s004.pdf]

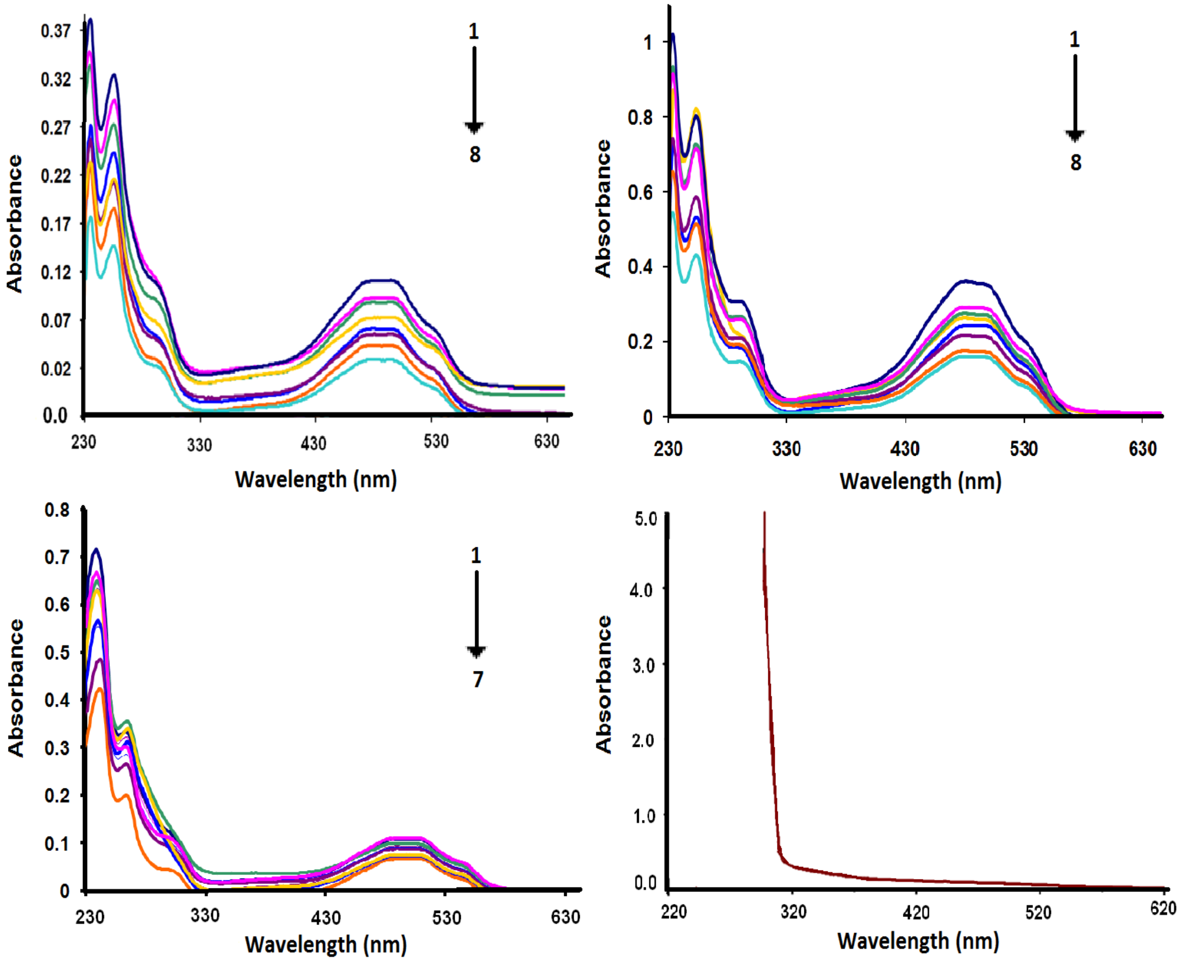

S4 Fig. UV-Visible signatures for the interaction in Mcllvaine buffer between DNA and 5.0μM of (A) DXH (B) Epi-DXH (C) DNR and (D) AA. {C_{DNA}=0, 2μM, 2.5μM, 3μM, 3.5μM, 4.0μM, 4.5 μM; 5.0 μM. Arrow direction indicated the increasing concentrations of DNA, pH =4.7, T=309.5K}.
