# Supplementary material for: Electrochemical, spectroscopic and theoretical monitoring of anthracyclines’ interactions with DNA and ascorbic acid by adopting two routes: Cancer cell line studies
Source: PLoS One. 2018 Oct 29;13(10):e0205764. doi: 10.1371/journal.pone.0205764 (PMC6205586; doi:10.1371/journal.pone.0205764)
Supplement: S5 Fig — (PDF) [file pone.0205764.s005.pdf]

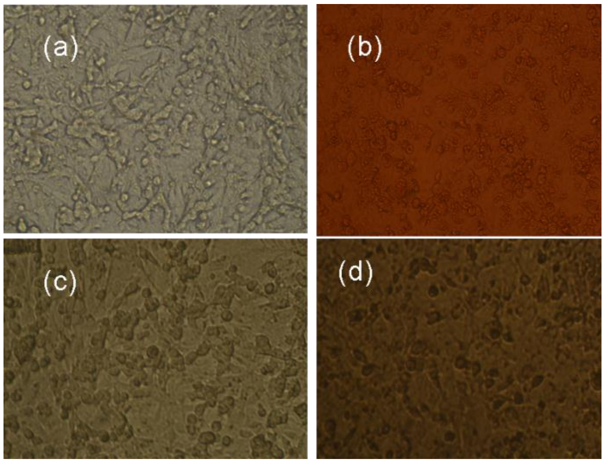

DXH

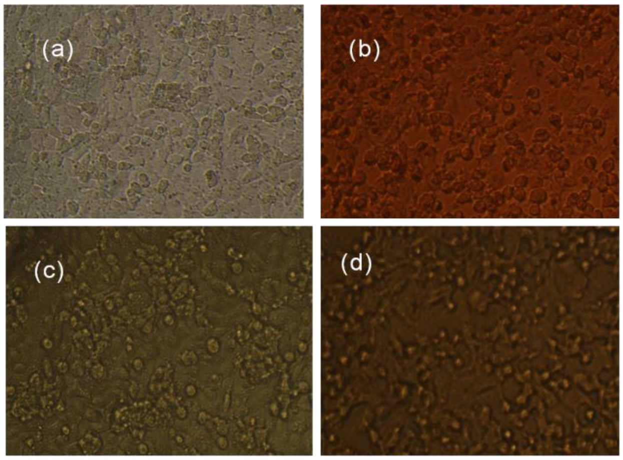

EpiDXH

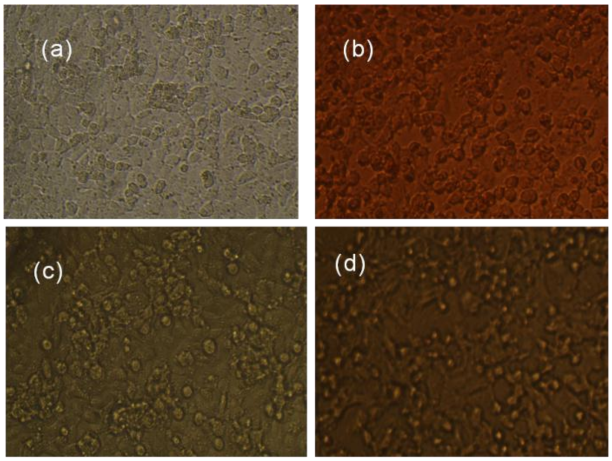

DNR

S5 Fig. (a) Control for cell line H-1299, **(b)** Intracellular drug distribution (2mM drug) and drug-induced cell damage, **(c)** Intracellular AA distribution (2mM AA) and AA induced cell damage, **(d)** Intracellular drug distribution (IC_{50}coadministerd with 2mM AA) and drug-induced cell damage
