# Supplementary material for: Electrochemical, spectroscopic and theoretical monitoring of anthracyclines’ interactions with DNA and ascorbic acid by adopting two routes: Cancer cell line studies
Source: PLoS One. 2018 Oct 29;13(10):e0205764. doi: 10.1371/journal.pone.0205764 (PMC6205586; doi:10.1371/journal.pone.0205764)
Supplement: S1 Table — (PDF) [file pone.0205764.s006.pdf]

S1 Table. Electrochemical parameters of DXH, EPiDXH and DNR in the absence and presence of different concentrations of DNA at pH 7.4 and 4.7{buffer: 0.12M Mcllvainesolution,Temp: 309.5K, Scan rate:0.1V/s.

pH 7.4							pH 4.7						
DXH–DNA													
[DNA] /µM	E_p^c/ V	E_p^a/ V	i_p^c/µA	i_p^a/µA	E_p^c-$E_{p/2}$/ V	$E^c_{1/2}$/ V	[DNA] /µM	E_p^c/ V	E_p^a/ V	i_p^c/µA	i_p^a/µA	E_p^c-$E_{p/2}$/ V	$E^c_{1/2}$/ V
0	-0.56	-0.52	5.82	4.99	0.060	-0.54	0	-0.50	-0.45	8.03	7.29	0.060	-0.49
2.0	-0.54	-0.50	5.12	4.63	0.060	-0.52	2.0	-0.49	-0.41	7.19	6.90	0.060	-0.47
2.5	-0.52	-0.48	3.42	3.82	0.059	-0.50	2.5	-0.47	-0.38	6.81	5.75	0.058	-0.42
3.0	-0.50	-0.46	4.34	2.92	0.060	-0.48	3.0	-0.42	-0.36	6.02	4.89	0.045	-0.39
3.5	-0.48	-0.44	4.25	2.89	0.060	-0.46	3.5	-0.39	-0.36	5.63	4.45	0.049	-0.36
4.0	-0.46	-0.42	3.23	1.80	0.060	-0.44	4.0	-0.35	-0.34	5.15	3.99	0.051	-0.31
4.5	-0.45	-0.42	2.52	0.72	0.060	-0.43	4.5	-0.32	-0.31	5.15	3.99	0.051	-0.30
EpiDXH–DNA													
0	-0.62	-0.45	4.31	4.26	0.090	-0.54	0	-0.60	-0.47	4.58	4.11	0.053	-0.54
2.0	-0.57	-0.45	3.69	3.75	0.074	-0.51	2.0	-0.57	-0.45	3.91	3.24	0.059	-0.42
2.5	-0.43	-0.37	2.99	2.95	0.068	-0.40	2.5	-0.54	-0.45	2.99	2.95	0.050	-0.50
3.0	-0.43	-0.40	2.92	2.82	0.067	-0.41	3.0	-0.52	-0.43	1.82	1.95	0.050	-0.49
3.5	-0.43	-0.43	0.97	0.94	0.068	-0.43	3.5	-0.49	-0.43	1.53	1.33	0.050	-0.45
DNR–DNA													
0	0.70	0.40	2.02	2.40	0.094	0.55	0	0.38	0.15	1.27	1.11	0.074	0.27
2.0	0.73	0.49	2.33	2.89	0.071	0.61	2.0	0.47	0.31	1.82	-2.00	0.103	0.36
2.5	0.75	0.51	2.26	2.76	0.078	0.63	2.5	0.47	p.d	1.74	p.d	0.110	0.36
3.0	0.75	0.50	2.09	1.97	0.078	0.63	p.d	p.d	p.d	p.d	p.d	p.d	p.d
3.5	0.76	0.50	1.47	1.33	0.064	0.63	p.d	p.d	p.d	p.d	p.d	p.d	p.d

p.d – Peak disappeared
