# Supplementary material for: Electrochemical, spectroscopic and theoretical monitoring of anthracyclines’ interactions with DNA and ascorbic acid by adopting two routes: Cancer cell line studies
Source: PLoS One. 2018 Oct 29;13(10):e0205764. doi: 10.1371/journal.pone.0205764 (PMC6205586; doi:10.1371/journal.pone.0205764)
Supplement: S2 Table — {buffer: 0.12M Mcllvaine solution, Temp: 309.5K, Scan rate: 0.1V/s. (PDF) [file pone.0205764.s007.pdf]

S2 Table. Electrochemical parameters of DXH–AA, EPiDEX–AA and DNR–AA in the absence and presence of different concentrations of DNA at pH 7.4 and 4.7. {buffer: 0.12M Mcllvaine solution, Temp: 309.5K, Scan rate: 0.1V/s.

pH 7.4							pH 4.7						
DXH–AA–DNA													
[DNA] /μM	E_p^c/ V	E_p^a/ V	i_p^c/μ A	i_p^a/ μA	E_p^c- $E_{p/2}$/V	$E^c_{1/2}$/ V	[DNA]/μM	E_p^c/ V	E_p^a/ V	i_p^c/μ A	i_p^a/μ A	E_p^c- $E_{p/2}$/ V	$E^c_{1/2}$/ V
0	-0.57	-0.53	3.42	2.92	-0.052	-0.55	0	-0.50	-0.45	8.03	7.29	0.060	-0.48
2.0	-0.57	-0.53	2.14	1.70	-0.054	-0.55	2.0	-0.48	-0.43	7.00	7.00	0.059	-0.46
2.5	-0.56	-0.52	2.00	2.11	-0.054	-0.54	2.5	-0.48	-0.43	6.10	6.99	0.059	-0.46
3.0	-0.55	-0.51	1.61	4.98	-0.054	-0.53	3.0	-0.48	-0.43	6.61	6.87	0.059	-0.46
3.5	-0.56	-0.52	1.48	5.02	-0.054	-0.54	3.5	-0.48	-0.43	5.58	5.80	0.059	-0.46
							4.0	-0.48	-0.43	4.70	4.91	0.059	-0.46
EpiDXH–AA–DNA													
0	-0.65	-0.48	4.64	4.61	0.075	-0.57	0	-0.58	-0.47	4.31	4.26	0.06	-0.54
2.0	-0.59	-0.52	3.69	3.75	0.067	-0.55	2.0	-0.47	-0.42	3.69	3.75	0.067	-0.46
2.5	-0.59	-0.52	2.95	2.99	0.074	-0.55	2.5	-0.45	-0.39	2.95	2.99	0.074	-0.42
3.0	-0.59	-0.52	2.73	2.81	0.068	-0.55	3.0	-0.45	-0.39	2.73	2.81	0.068	-0.42
DNR–AA–DNA													
0	0.66	0.47	2.33	2.89	0.084	0.55	0	0.39	0.27	3.86	3.41	0.040	0.33
2.0	0.64	0.46	2.12	2.84	0.082	0.55	2.0	0.36	0.33	4.64	2.21	0.035	0.34
2.5	0.64	0.45	1.97	2.79	0.071	0.55	2.5	0.38	0.35	3.75	2.85	0.039	0.35
3.0	0.64	0.44	2.26	2.76	0.078	0.55	3.0	0.38	0.33	3.30	2.07	0.039	0.34
3.5	0.63	0.44	2.09	1.97	0.078	0.54	3.5	0.38	0.33	3.18	1.68	0.040	0.34
