# Supplementary material for: Electrochemical, spectroscopic and theoretical monitoring of anthracyclines’ interactions with DNA and ascorbic acid by adopting two routes: Cancer cell line studies
Source: PLoS One. 2018 Oct 29;13(10):e0205764. doi: 10.1371/journal.pone.0205764 (PMC6205586; doi:10.1371/journal.pone.0205764)
Supplement: S3 Table — (PDF) [file pone.0205764.s008.pdf]

**S3 Table.** Binding parameters obtained from Cyclic Voltammetricand UV-Vis spectroscopic studies at pH 4.7and T=309.5K

| | Experimental | | | | |
|---|---|---|---|---|---|
| | Cyclic Voltammetric | | | UV-Vis spectroscopic | |
| complexes | $K_b$/M$^{1}$ ×10$^{5}$ | -ΔG/kJmol$^{-1}$ | Binding site size (s)/bp | $K_b$/M$^{-1}$ ×10$^{5}$ | -ΔG/kJmol$^{-1}$ |
| Binary complexes | | | | | |
| AA-DNA | 1.31 | 30.31 | 2.00 | 0.02 | 19.55 |
| DXH-DNA | 3.30 | 32.61 | 1.60 | 0.17 | 25.06 |
| EpiDXH-DNA | 0.45 | 27.57 | 1.31 | 0.14 | 24.56 |
| DNR-DNA | 19.2 | 37.22 | 1.51 | 11.8 | 35.97 |
| Binary complexes | | | | | |
| DXH-AA | 0.083 | 23.22 | ---- | 0.74 | 32.99 |
| EpiDXH-AA | 0.001 | 11.19 | ---- | 0.013 | 17.77 |
| DNR-AA | 0.005 | 21.76 | ---- | 0.02 | 19.68 |
| Route-1 Tertiary complexes | | | | | |
| DXH-AA-DNA | 1.15 | 30.19 | 0.58 | 2.51 | 31.99 |
| EpiDXH-AA-DNA | 1.75 | 31.06 | 0.50 | 2.75 | 32.23 |
| DNR-AA-DNA | 21.1 | 37.47 | 2.14 | 2.13 | 31.57 |
| Route-2 Tertiary complexes | | | | | |
| DXH-DNA-AA | 0.93 | 29.43 | 0.37 | 1.17 | 30.02 |
| EpiDXH-DNA-AA | 1.01 | 29.65 | 0.19 | 1.03 | 29.7 |
| DNR-DNA-AA | 7.32 | 34.74 | 0.22 | 1.14 | 29.96 |
